# New Research on Childbirth Has the Potential to Empower Women's Decision Making, but More Is Needed

**DOI:** 10.1371/journal.pmed.1001197

**Published:** 2012-03-27

**Authors:** The PLoS Medicine Editors

## Abstract

The *PLoS Medicine* editors discuss new research studies on the risks associated with mode of childbirth following caesarean section.

This month, *PLoS Medicine* publishes two research articles with new data that should help women and clinicians make better informed decisions about childbirth [1],[2]. The two articles are further discussed in a commentary [3]. These studies touch on a highly emotive and controversial area—the risks associated with planned mode of birth. The research findings stay well away from recent debates, widely publicized in the media, around whether a woman has the right to a caesarean section on request [4],[5]. Rather, the studies provide valuable new estimates of the specific risks of measures of infant and maternal morbidity and fetal or liveborn infant death associated with either planned vaginal birth or planned caesarean section, for women who have had a previous caesarean section delivery.

One study [1], conducted by Caroline Crowther and colleagues in Australian maternity hospitals, aimed to compare the benefits and risks of planned vaginal birth versus planned elective repeat caesarean section following a previous caesarean section. The study enrolled just over 2,000 pregnant women who had had a single previous caesarean section delivery and were judged eligible to deliver the current pregnancy either vaginally or by repeat caesarean section. Critically, the researchers found that the risks of very severe outcomes—such as fetal or infant death—were lower among women who planned a repeat caesarean section than among women who planned a vaginal birth. However, the absolute differences in risk of such serious outcomes between the two groups were very small. It is notable that although the investigators attempted to conduct a randomized trial as part of this study, very few women consented to be randomized to the two alternative modes of delivery. So the majority of women in the study constituted a “preference cohort,” giving birth according to their intended mode, or according to the clinical decision ultimately made following their initial preference. Randomized trials are generally considered to be the “gold standard” for research aiming to deliver robust evidence on the effects of interventions, but clearly in many clinical situations in which women have strong preferences—such as this one—a randomized study is difficult or impossible to carry out. However, despite this important caveat, the study is highly pragmatic, defined as research that is “primarily designed to determine the effects of an intervention under the usual conditions in which it will be applied” [6]. This is particularly true in relation to one dimension of pragmatism, that of compliance with the intervention originally planned (a pragmatic study would essentially ignore deviations from planned, or assigned, intervention).The study by Crowther and colleagues is one of the few in this area providing evidence on the outcomes associated with *planned*, as opposed to actual, birth mode, which therefore helps clinicians to counsel women on the outcomes associated with their choice.

The second study [2] examines the risk of a very rare and serious outcome in childbirth: uterine rupture. Fitzpatrick and colleagues collected data for all cases of uterine rupture in the UK that occurred between April 2009 and April 2010. The researchers then examined predictors for this event and showed that the risk of uterine rupture is higher among women who have had two or more previous caesarean sections, and if the time period since the last caesarean section is less than 12 months. These data provide a counterpoint to the findings of the study by Crowther and colleagues [1] in that it cautions women who wish for a larger family about the risk of certain, albeit very rare, outcomes associated with multiple repeat caesarean sections.

The bigger issue raised by these findings relates to how clinicians and women can work together to make the best possible decision when so many questions remain unanswered. The existing research presents an incomplete picture of the entire set of possible risks associated with the options that women and clinicians have; under-studied issues relate to the implications not just for *this* birth, but for subsequent births too. For example, as highlighted by Crowther and colleagues [1], much longer-term follow-up, and more knowledge about the risks arising from multiple caesarean sections, are needed. Together, these findings highlight the importance of pragmatic research studies, with the overall goal of improving care for future generations of mothers and babies. The long list of questions for further examination, and the limitations inherent in both studies, demonstrate that improving the evidence base will not be easy.
